# Tailoring the effect of antithrombin-targeting therapy in haemophilia A using in silico thrombin generation

**DOI:** 10.1038/s41598-021-95066-8

**Published:** 2021-07-30

**Authors:** Romy M. W. de Laat-Kremers, Marisa Ninivaggi, Iris van Moort, Moniek de Maat, Bas de Laat

**Affiliations:** 1 Department of Data Analysis and Artificial Intelligence, Synapse Research Institute, Pastoor Habetsstraat 50, 6217 KM, Maastricht, The Netherlands; 2 Department of Functional Coagulation, Synapse Research Institute, Maastricht, The Netherlands; 3 Department of Hematology, Erasmus University Medical Center, Rotterdam, The Netherlands

**Keywords:** Biomarkers, Experimental models of disease, Translational research, Drug regulation, Drug therapy

## Abstract

Factor (F) VIII deficiency causes bleeding in haemophilia A patients because of the reduced formation of procoagulant enzyme thrombin, which is needed to make the blood clot. We measured the dynamics of coagulation in haemophilia A patients by measuring thrombin generation (TG). Additionally, we quantified the procoagulant process of prothrombin conversion and anticoagulant process of thrombin inhibitor complex formation. In haemophilia A, prothrombin conversion is severely reduced, causing TG to be low. Nevertheless, the thrombin inactivation capacity of these patients is comparable to that in healthy subjects, leading to a severe imbalance between procoagulant and anticoagulant processes and a subsequent increased bleeding risk. A novel therapy in haemophilia A is the targeting of anticoagulant pathway, e.g. thrombin inhibitor antithrombin (AT), to restore the haemostatic balance. We simulated the effect of AT reduction on TG in silico. Lowering AT levels restored TG dose-dependently and an AT reduction of 90–95% led to almost normal TG in most patients . However, the variation in response to AT reduction was large between patients, indicating that this approach should be tailored to each individual patients. Ideally, TG and thrombin dynamics simulation could in the future contribute to the management of patients undergoing AT targeting therapy.

Haemophilia A is a hereditary bleeding disorder caused by the partial or complete deficiency of coagulation factor VIII (FVIII), which affects 1 in 5000 males^1^. Patients with haemophilia A are categorized into mild (0.05–0.4 IU/mL FVIII), moderate (0.01–0.05 IU/mL FVIII) or severe (< 0.01 IU/mL FVIII) cases according to their plasma FVIII level. The bleeding phenotype of these patients ranges from mild (prolonged bleeding after tooth extraction, trauma or surgery) to severe (spontaneous joint and muscle bleeding), although this does not always correspond with the FVIII plasma level^2^.

The thrombin generation (TG) test is a global coagulation assay, which measures the overall function of the coagulation system. In general, a high TG test result is associated with thrombosis^3–5^, whereas low TG is related to bleeding problems^6–8^. The TG test has previously been used to evaluate the haemostatic potential in haemophilia A patients and proved to be especially helpful for treatment management in these patients^9^. Typically, the TG test result is reduced in haemophilia A patients, but not fully absent^10^, and can be attributed to an imbalance in the pro- and anticoagulants in haemophilia A patients. Activated FVIII is the essential cofactor of FIXa, which converts FX into FXa. FXa together with FVa subsequently activates prothrombin into thrombin, allowing the formation of a fibrin clot. The production of thrombin is impeded in haemophilia A due to low levels of FVIII, which plays an important role in the acceleration of thrombin production. This causes the haemostatic balance to shift towards an over-anticoagulated system, which is associated with bleeding, because thrombin inactivation mechanisms remain unchanged in haemophilia A.

Thrombin dynamics analysis quantifies the processes of prothrombin conversion and thrombin inactivation that determine the balance between hypo- and hypercoagulability^11^. It can be used to accurately pinpoint a specific mechanistic target in the coagulation cascade and thus study underlying mechanisms of the coagulation system. In haemophilia A, the reduction of TG caused by low FVIII activity attenuates the conversion of prothrombin into thrombin. Prothrombin conversion is usually not routinely determined in haemophilia A. Nevertheless, the quantification of prothrombin conversion can give insight in both the effect of lower FVIII levels and the role of the remainder of the coagulation system, which is known to vary a lot between individuals, and its interplay with the low FVIII levels.

Currently, FVIII concentrates are used to treat bleeding in severe haemophilia prophylactically or therapeutically. Despite the success of FVIII replacement therapy, the treatment is invasive and stressful for the patients, because intravenous infusion is needed two to three times a week. Therefore, new treatment strategies for haemophilia A have been designed. A novel treatment approach is the restoration of the balance between pro- and anticoagulants, which is distorted in haemophilia A due to the lack of FVIII. Haemophilia A patients that suffer from co-inherited deficiencies in natural anticoagulants, such as antithrombin (AT) or protein C and S have a favourable bleeding phenotype compared to haemophilia patients without co-inherited deficiencies^12–16^. Additionally, a murine model showed that TG was increased upon AT reduction^17^. Fitusiran is a novel RNAi therapeutic that mimics this protective trait by reducing the production of the natural anticoagulant AT in haemophilia A patients^18–20^. This approach aims to restore to restore the balance between pro- and anticoagulant pathways that determine the TG process, by lowering thrombin inactivation as a response to reduced prothrombin conversion due to the lack of FVIII.

In this study, we investigated the balance of prothrombin conversion and thrombin inactivation in mild, moderate and severe haemophilia A patients and healthy controls and studied the inter-individual variability. In addition, we used in silico modelling to investigate the effect of AT reduction on the TG curves and the haemostatic balance in haemophilia A patients.

## Results

### Thrombin generation

**Figure 1:**
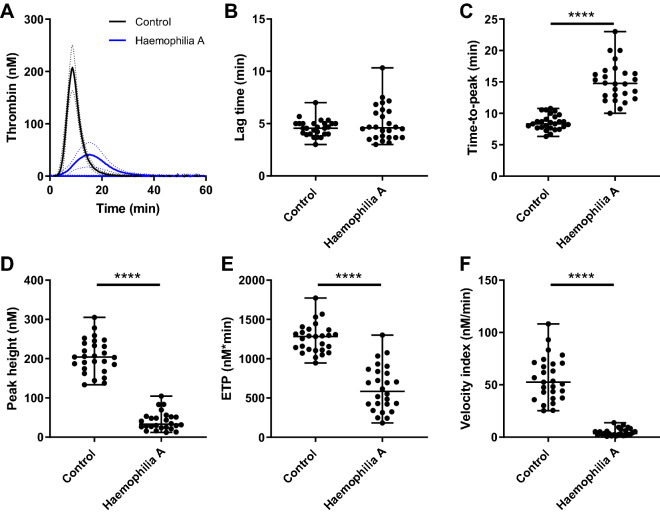
Thrombin generation in haemophilia A triggered with 1 pM tissue factor. TG was triggered with 1 pM TF and the average curves for the patient and control group are shown in (**A**). Individual TG curves were quantified by the lag time (**B**), time-to-peak (**C**), peak height (**D**), endogenous thrombin potential (**E**) and velocity index (**F**). TG curves are depicted as the mean curve (continuous line) ± the 95% confidence interval (dotted lines) and the parameter values are shown as the individual values with the median ± interquartile range. Statistical significance according to the Mann Whitney test is indicated as **** for a *p*-value < 0.001.

**Figure 2:**
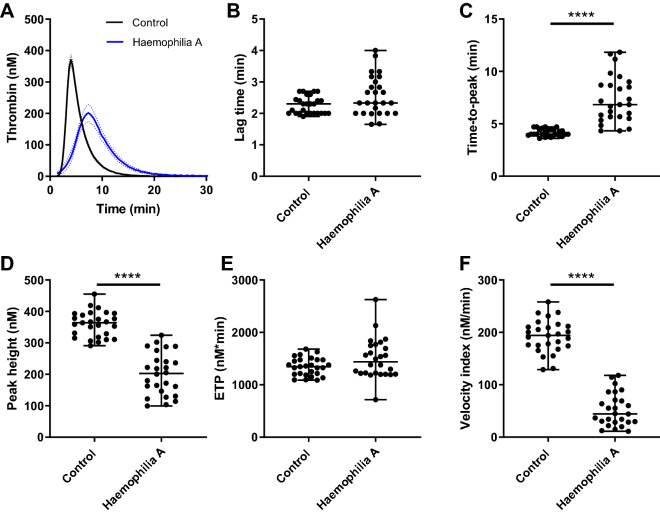
Thrombin generation in haemophilia A triggered with 5 pM tissue factor. TG was triggered with 5 pM TF and the average curves for the patient and control group are shown in (**A**). Individual TG curves were quantified by the lag time (**B**), time-to-peak (**C**), peak height (**D**), endogenous thrombin potential (**E**) and velocity index (**F**). TG curves are depicted as the mean curve (continuous line) ± the 95% confidence interval (dotted lines) and the parameter values are shown as the individual values with the median ± interquartile range. Statistical significance according to the Mann Whitney test is indicated as **** for a *p*-value < 0.001.

TG was measured in 26 haemophilia A patients and 27 age- and sex-matched controls (Table 1). The mean age for both groups was 49 years and the average FVIII level was 0.083 IU/mL and 1.41 IU/mL, in patients and controls respectively. In the haemophilia A group, based on the FVIII levels, 11 patients were classified as mild haemophilia A patients, 10 patients as moderate and 5 patients as severe. The TG test was triggered with 1 pM TF (Fig. 1) and 5 pM tissue factor (TF) (Fig. 2). The average TG curve of the haemophilia A patients is distinctly lower than the average TG curve of the control group for both TF concentrations. Time-to-peak was prolonged by 1.8-fold (*p* < 0.001) in haemophilia A patients. Peak height (-80%), velocity index (− 92%) and ETP (− 51%) were significantly lower in patients (*p* < 0.001) at 1 pM TF. Similarly, at 5 pM TF, peak height and velocity index were significantly lower in patients (− 44% and − 73%, *p* < 0.001), whereas, the ETP did not differ between patients and controls (1344 nM*min vs. 1498 nM*min). The lag time did also not differ between the two groups, irrespectively of the trigger concentration. TG results showed a trend towards lower peak height according to the severity of haemophilia A, but this was not statistically significant.

**Table 1:** General characteristics and thrombin generation and thrombin dynamics data of controls and haemophilia A patients.

	Controls	Haemophilia A	Controls versus patients *p*-value	Mild	Moderate	Severe	Severity *p*-value
Number	27	26		11	10	5	
**General characteristics**							
Sex, % male	100%	100%		100%	100%	100%	
Age (± SD)	48.7 ± 9.32	49.3 ± 19.46	*ns*	47.6 ± 17.0	55.3 ± 22.1	40.0 ± 17.7	*ns*
FVIII (IU/mL)	1.41 ± 4.4	0.08 ± 0.10	< *0.001*	0.16 ± 0.11	0.03 ± 0.01	< 0.01	< *0.001*
Fibrinogen (g/L)	3.3 ± 1.0	5.2 ± 1.7	< *0.001*	5.3 ± 1.6	5.2 ± 1.9	4.9 ± 1.8	*ns*
**Thrombin generation—1 pM tissue factor**							
Lag time (min)	4.6 ± 0.8	5.0 ± 1.7	*ns*	5.3 ± 2.0	4.9 ± 1.3	4.8 ± 2.1	*ns*
Peak height (nM)	206 ± 43	41 ± 24	< *0.001*	45 ± 27	40 ± 24	34 ± 17	*ns*
Time-to-peak (nM)	8.5 ± 1.2	15.0 ± 3.1	< *0.001*	14.8 ± 2.4	14.9 ± 3.0	15.8 ± 4.8	*ns*
ETP (nM*min)	1258 ± 187	620 ± 291	< *0.001*	663 ± 324	606 ± 295	550 ± 240	*ns*
Velocity index (nM/min)	56 ± 21	4.7 ± 3.4	< *0.001*	5.4 ± 3.9	4.5 ± 3.4	3.7 ± 2.6	*ns*
**Thrombin generation—5 pM tissue factor**							
Lag time (min)	2.3 ± 0.3	2.6 ± 0.6	*ns*	2.7 ± 0.5	2.5 ± 0.6	2.4 ± 1.0	*ns*
Peak height (nM)	360 ± 41	202 ± 67	< *0.001*	219 ± 68	191 ± 63	184 ± 79	*ns*
Time-to-peak (nM)	4.1 ± 0.3	7.3 ± 2.2	< *0.001*	7.2 ± 1.5	7.4 ± 2.6	7.5 ± 3.3	*ns*
ETP (nM*min)	1344 ± 169	1498 ± 382	*ns*	1644 ± 473	1422 ± 316	1329 ± 136	*ns*
Velocity index (nM/min)	192 ± 31	52 ± 32	< *0.001*	54 ± 29	51 ± 33	52 ± 41	*ns*
**Thrombin dynamics—1 pM tissue factor**							
Total prothrombin conversion (nM)	619 ± 106	330 ± 154	< *0.001*	327 ± 140	335 ± 172	330 ± 154	*ns*
Maximum prothrombin conversion rate (nM/min)	169 ± 68	27 ± 14	< *0.001*	29 ± 14	26 ± 14	27 ± 14	*ns*
Thrombin-antithrombin complexes (nM)	598 ± 105	300 ± 147	< *0.001*	229 ± 135	301 ± 159	300 ± 147	*ns*
Thrombin-α2-macroglobulin complexes (nM)	24 ± 7	16 ± 9	< *0.001*	15 ± 7	19 ± 12	16 ± 9	*ns*
**Thrombin dynamics—5 pM tissue factor**							
Total prothrombin conversion (nM)	892 ± 147	755 ± 205	*0.004*	769 ± 199	756 ± 224	755 ± 205	*ns*
Maximum prothrombin conversion rate (nM/min)	484 ± 65	154 ± 69	< *0.001*	149 ± 59	163 ± 72	154 ± 69	*ns*
Thrombin-antithrombin complexes (nM)	856 ± 142	703 ± 198	*0.012*	722 ± 197	697 ± 217	703 ± 198	*ns*
Thrombin-α2-macroglobulin complexes (nM)	38 ± 12	41 ± 13	*n.s*	40 ± 6	48 ± 18	41 ± 13	*ns*
Thrombin decay capacity (min^−1^)	0.712 ± 0.089	0.520 ± 0.134	< *0.001*	0.497 ± 0.122	0.536 ± 0.138	0.541 ± 0.171	*ns*

Data is shown as mean ± standard deviation for the control and combined patient group. Statistical differences between the control and combined patient group are indicated as a *p*-value below 0.05 according to the Mann–Whitney test. Data is shown as mean ± standard deviation for subgroups of the patient group. Statistical differences between the subgroups are indicated as a *p*-value below 0.05 according to the Kruskal–Wallis test.

### Thrombin dynamics

**Figure 3:**
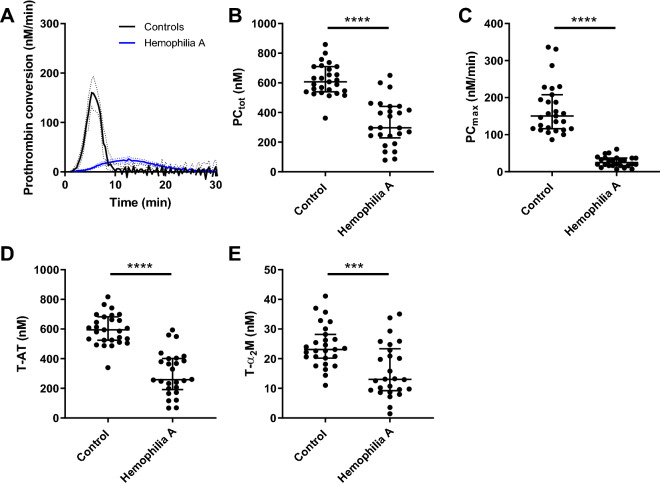
Prothrombin conversion in haemophilia A triggered with 1 pM tissue factor. Prothrombin conversion curves were determined from TG curves measured at 1 pM TF. (**A**) Average prothrombin conversion curves for the patient and control group. Individual TG curves were quantified by the total prothrombin conversion (PC_tot_, **B**), maximum prothrombin conversion rate (PC_max_, **C**), thrombin-antithrombin complex formation (T-AT, **D**), and thrombin-α_2_macroglobulin complex formation (T-α_2_M) (**E**). Prothrombin conversion curves are depicted as the mean curve (continuous line) ± the 95% confidence interval (dotted lines) and the parameter values are shown as the individual values with the median ± interquartile range. Statistical significance according to the Mann Whitney test is indicated as *** for a *p*-value < 0.001, **** for a *p*-value < 0.0001.

**Figure 4:**
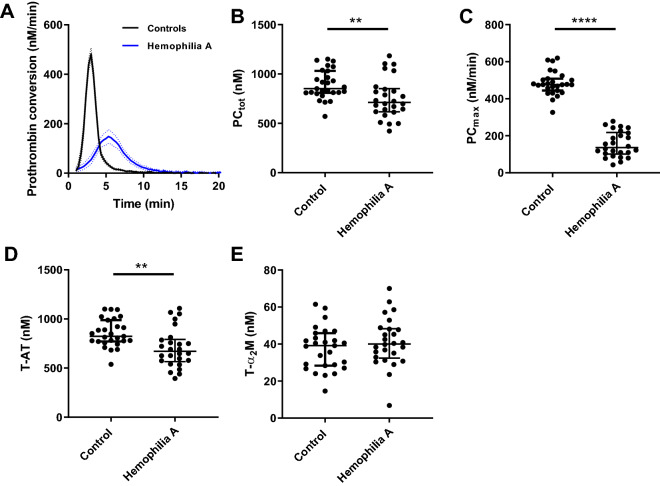
Prothrombin conversion in haemophilia A triggered with 5 pM tissue factor. Prothrombin conversion curves were determined from TG curves measured at 5 pM TF. (**A**) Average prothrombin conversion curves for the patient and control group. Individual TG curves were quantified by the total prothrombin conversion (PC_tot_, **B**), maximum prothrombin conversion rate (PC_max_, **C**), thrombin-antithrombin complex formation (T-AT, **D**), and thrombin-α_2_macroglobulin complex formation (T-α_2_M) (**E**). Prothrombin conversion curves are depicted as the mean curve (continuous line) ± the 95% confidence interval (dotted lines) and the parameter values are shown as the individual values with the median ± interquartile range. Statistical significance according to the Mann Whitney test is indicated as ** for a *p*-value < 0.01, **** for a *p*-value < 0.0001.

TG is a balance between pro- and anticoagulant processes, and when this balance is disturbed, it will result in bleeding or thrombosis. The pro- and anticoagulant processes underlying TG can be studied computationally by thrombin dynamics analysis^11^. In this approach, prothrombin conversion is quantified by the total amount of prothrombin converted (PC_tot_) and the maximum prothrombin conversion rate (PC_max_). Prothrombin conversion was calculated from the TG curves measured at 1 and 5 pM TF (Figs. 3 and 4). The average prothrombin conversion curve was lower in haemophilia A patients than in controls. The quantification of each individual curve showed that both PC_tot_ and PC_max_ are significantly lower in the haemophilia group (*p* < 0.001), independently of the TF concentration. In patients and controls, PC_max_ was significantly correlated with the plasma FVIII level: R = 0.85 with *p* < 0.001 at 1 pM TF and R = 0.75 with *p* < 0.001. Interestingly, at 1 pM TF, the FVIII level was positively correlated with PC_tot_ (R = 0.48, *p* < 0.001), whereas the correlation was negative at 5 pM TF (R = − 0.69, *p* < 0.001). Thrombin inactivation during the TG test was quantified as the formation of thrombin-antithrombin (T-AT) and thrombin-α_2_Macroglobulin (T-α_2_M) complexes. T-AT and T-α_2_M were reduced by 50% and 33%, respectively in haemophilia patients compared to healthy controls (*p* < 0.0001) at 1 pM TF (Fig. 3D, E). Alike PC_tot_, T-AT and T-α_2_M complex formation was weakly positively associated with the FVIII level at low TF (R = 0.52 and R = 0.32, *p* < 0.05), but negatively associated at a higher TF concentration (R = − 0.68 and R = − 0.65, *p* < 0.001). Additionally we found a positive correlation between FVII and the thrombin decay capacity (R = 0.45, *p* < 0.001), indicating a higher thrombin inactivation capacity in subjects with higher FVIII levels.

### In silico experiments

**Figure 5:**
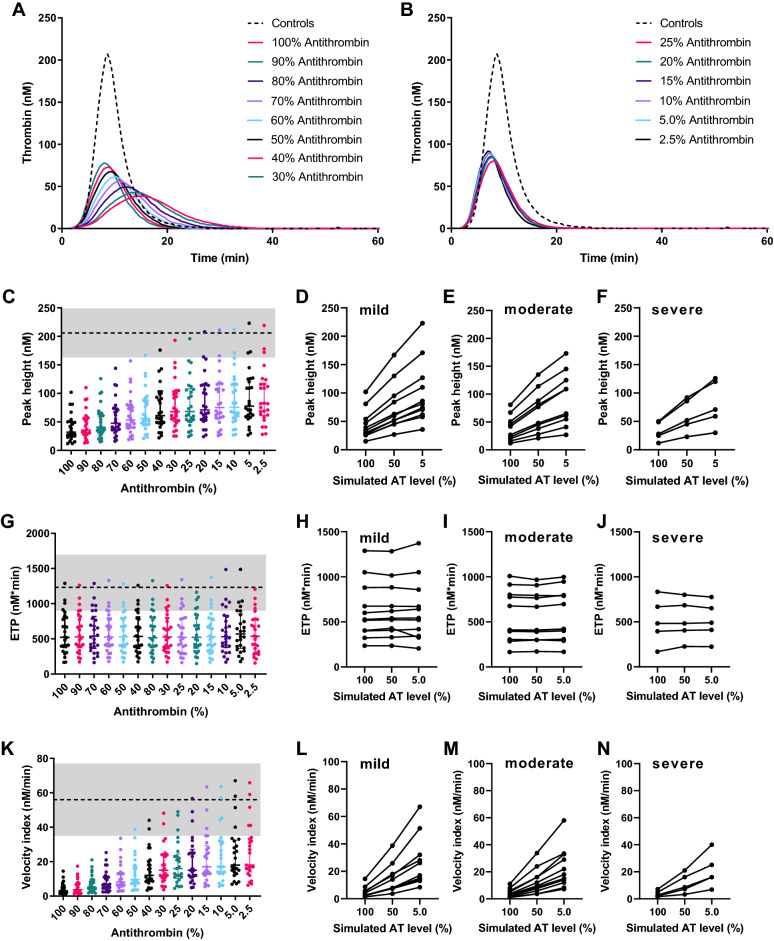
The in silico effect of antithrombin targeting in haemophilia on thrombin generation. (**A**) The average TG curve of the haemophilia A patients at the original plasma AT level (100%) in silico generated average thrombin generation curves of the same patients at 90, 80, 70, 60, 50, 40, and 30% AT. (**B**) The average in silico generated average thrombin generation curves of the haemophilia A patients at 25, 20, 15, 10, 5, and 2.5% AT. The generated TG curves were quantified by their peak height (**B**–**F**), ETP (**G**–**J**) and velocity index (**K**–**N**). References ranges in the healthy subject group are plotted as grey box with the mean value in healthy subjects indicated as a dashed line. The effect AT reduction to 50% and 5% of the original plasma level is depicted for the peak height for mild (**D**), moderate (**E**) and severe (**F**) haemophilia A patients, and the same is shown for the ETP (**H**–**J**) and the velocity index (**L**–**N**).

Lower TG in haemophilia A patients is caused by lower prothrombin conversion as a result of the (partial) deficiency in FVIII activity, while thrombin inactivation is unchanged. Targeting thrombin inactivation, more specifically thrombin inactivation by AT, has been proposed as a novel treatment strategy in haemophilia A^20^. We used in silico experimentation to simulate the reduction of AT in haemophilia A patients. We investigated whether targeting of antithrombin could restore the TG potential by lowering AT levels to 90%, 80%, 70%, 60%, 50%, 40%, 30%, 25%, 20%, 15%, 10%, 5% and 2.5% of the original plasma level (Fig. 5). The lowering of AT partially normalized the TG curve dose-dependently in all patients. Peak height and velocity index increased dose-dependently, ETP and lag time lag time were unchanged and time-to-peak decreased with decreasing AT level. The average peak height was 41 nM ± 24 nM in haemophilia patients at 100% AT. A reduction of 50% of the original AT level could restore the TG profile in haemophilia A patients up to an average peak height of 69 nM ± 37 nM. A further reduction of AT to 5% increased the peak height to 93 ± 48 nM. For some patients, the peak height was restored to be within the normal range if AT levels were below 15%.

Moreover, the mean velocity index of 4.6 nM/min ± 3.3 nM/min at 100% AT in haemophilia A patients was restored to 13.5 nM/min ± 9.4 nM/min at 50% antithrombin. The reduction of AT to 5% increased the velocity index to 24.5 ± 15.9 nM/min and thereby reached the normal range in some patients.

Figure 5 shows that the effect of AT lowering varies between the patients, irrespectively of their FVIII plasma level. If AT was reduced to 50%, this resulted in an average increase of the peak height by 56% in mild haemophilia A patients, 75% in moderate patients and 82% in severe patients. A further reduction to 5% AT, as is typically achieved with current therapeutic strategies increased the peak height by 109%, 136%, and 148%, respectively in mild, moderate and severe haemophilia A patients.

## Discussion

TG is known to be reduced in haemophilia A because low FVIII levels attenuate the activation pathway of prothrombin. In the present study, we quantified prothrombin conversion and thrombin inactivation in haemophilia A using thrombin dynamics analysis^11^ and investigated inter-patient variation. Additionally, we studied the effect of novel AT targeting treatment strategies on TG using in silico experimentation^21^.

TG parameters ETP and peak height were significantly decreased in haemophilia A patients, especially when triggered with a low TF concentration. The effect on the ETP was less pronounced when TG was triggered with higher TF concentration, probably because then the assay becomes less sensitive to the factors of the intrinsic coagulation cascade^22^. Nevertheless, thrombin was formed at a slower pace, which was reflected in a longer time-to-peak and a lower peak height and velocity index. Thrombin dynamics analysis revealed that prothrombin conversion is decreased in haemophilia A patients. PC_tot_ was significantly lower in haemophilia A patients and also this effect is more pronounced at low TF concentrations. PC_max_ was significantly lower in patients for both triggers, indicating that the TG profile is not only lower because less thrombin is formed but also because it is formed at a slower rate. As a consequence, T-AT and T-α_2_M complex formation, which depends on the amount of thrombin formed, are lower in haemophilia A.

Our results can be explained by the (partial) FVIII deficiency in haemophilia A, resulting in decreased prothrombin conversion downstream the coagulation cascade when a low TF trigger is used as previously reported^23^. FVIII is an important cofactor in the coagulation cascade that, once activated, can enhance the velocity of FXa formation by FIXa in vitro, up to 10^3^–10^6^-fold (depending of the experimental conditions used)^24^. Moreover, irrespectively of the trigger concentration used, no differences in lag time were observed between patients and controls. This is mainly due to the initiation of the TG test with TF, which together with FVIIa can activate FX and subsequently form the first traces of thrombin independently of FVIII (a)^25^.

We show that the balance between pro- and anticoagulant shifts towards bleeding in haemophilia A. It has been proposed in the past that the balance between prothrombin conversion and thrombin inactivation can be restored by decreasing the inactivation of the formed thrombin, e.g. by inhibiting APC, AT or TFPI^26,27^. The inhibition of AT in silico increases TG test parameters ETP, peak height and velocity index significantly, suggesting that targeting AT could be successful in haemophiliacs in an attempt to restore the balance between prothrombin conversion and thrombin inactivation. Pasi et al. recently showed in a phase I study that targeting AT with RNAi therapeutical fitusiran increased TG in both haemophilia A and B patients in a dose-dependent manner^20^. Our results suggest that a large reduction of AT is necessary to reach the thrombin generation capacity of a normal subject. Indeed, the phase I study on fitusiran indicated that a therapeutical reduction of antithrombin levels by 75% in hemophilia A patients brings the thrombin generation capacity into the lower normal range^20^. The phase II study on fitusiran showed that an AT reduction of 78–88% was achieved when a fixed dose of 50 mg was administered^28^. Indeed our in silico analysis shows a reduction of AT by more than 85% bring TG in the normal range in a part of the patients, but not all.

Besides the variation in AT lowering after administration of a fixed dose of an antithrombin level reducing drug, our study indicates that inter-individual variation in response to AT reduction is large. The remainder of the coagulation system does not respond identically in each patient at a certain AT level and we show that the effect of AT reduction is irrespective of the baseline FVIII plasma level. Additionally, previous reports suggest that haemophilia treatment should become more individualized^27,29^. It would be interesting to investigate further how the TG test and thrombin dynamics analysis could be utilized to optimize the individual treatment of each patient. We now show for the first time that in silico thrombin dynamics experimentation could be used for example to estimate the dose of an AT-targeting drug in each individual patient. In a broader sense, the TG test and thrombin dynamics analysis might be used as a strategy to estimate the optimal dosing of other therapeutics such as FVIII or bypassing agents in the future.

There were some limitations in the current study, such as the low number of patients. Analysis of a higher number hemophilia A patients could render significant differences between mild, moderate and severe patient as well, in addition to the differences found between patients and controls. Additionally, the same methodology as used in the current study could be used to investigate the dynamics of thrombin generation in hemophilia B patients. Nevertheless, for this initial manuscript, we focused on hemophilia A because of its higher prevalence.

To conclude, we found a disturbed balance between prothrombin conversion and thrombin inactivation in patients with haemophilia A. Especially the rate of prothrombin conversion is lower in haemophilia patients causing low TG. Although PC_tot_ ranged from low to normal in patients compared to healthy subjects, thrombin formation in haemophilia A is a slower process due to the (partial) lack of FVIII. In silico experimentation shows that the therapeutic reduction of AT levels leads to an increased TG. AT reduction is a novel and successful treatment to prevent bleeding for haemophilia A patients. This study shows that thrombin generation and thrombin dynamics analysis are an opportunity to individualize patient care in the future bytailoring the dosing of AT level reducing drugs to the coagulation system of individual patients.

## Methods

### Participants and sample handling

26 haemophilia A patients and 27 age- and sex-matched control subjects were enrolled in this study (Table 1). Haemophilia A severity was determined based on the patients FVIII level: patients with FVIII levels below 0.40 IU/mL were classified as mild, below 0.05 IU/mL as moderate and below 0.01 IU/mL as severe haemophilia patients. Healthy subjects did not report any known coagulation defects and had FVIII levels above 0.85 IU/mL. The study protocol was approved by the medical ethics committees of the Erasmus Medical Center (Rotterdam) and the Maastricht University Medical Center (Maastricht). Written informed consent was obtained in concordance with the declaration of Helsinki. Whole blood was collected on 3.2% citrate in a 9:1 ratio for the preparation of platelet poor plasma (PPP), which was prepared consecutively by centrifuging twice at 2821 g for 10 min and then stored at  − 80 °C until further use. All methods were performed in accordance with the relevant guidelines and regulations.

### Thrombin generation

Calibrated Automated Thrombinography (CAT) was performed as previously described^30^. All wells contained 80 µl plasma and 20 µl tissue factor (TF; 1 or 5 pM; Innovin, Dade-Behring, Marburg, Germany) and phospholipids (4 µM; Synapse Research Institute, Maastricht, the Netherlands;^31^) or 20 µl of calibrator (100 nM; Synapse Research Institute, Maastricht, the Netherlands; ^30^). Thrombin generation (TG) was initiated by the addition of 20 µl of ZGGR-AMC (417 µM; Bachem, Bubendorf, Switzerland) and CaCl_2_ (16.7 mM).

The TG fluorescence data were converted to TG curves, as described elsewhere^32^ and further used to perform additional computational thrombin dynamics analysis to extract prothrombin conversion and quantify thrombin inactivation. The TG curves were quantified by the parameters lag time, time-to-peak, peak height, endogenous thrombin potential (ETP) and velocity index.

### Coagulation factor levels

Plasma FVIII, fibrinogen and AT levels were determined on the STA-R analyser, using respectively, STA ImmunoDef VIII with STA-CK Prest, STA Liquid Fibrinogen, and STA—Stahrom AT III and according to the manufacturer’s specifications (Diagnostica Stago, Asnières-sur-Seine, France). α_2_-macroglobulin (α_2_M) was measured in a functional assay (Synapse Research Institute, Maastricht, the Netherlands)^11^. AT, α_2_M and fibrinogen levels were used to perform thrombin dynamics analysis.

### Thrombin dynamics

The TG curve is the net result of prothrombin conversion and thrombin inactivation. Therefore, the course of prothrombin conversion can be calculated if thrombin formation and inactivation are known. Thrombin inactivation was determined by the previously described computational model which is based on the plasma AT, α_2_M and fibrinogen level, according to Eqs. (1)–(4).

1 $$ {\text{d}}\left( {{\text{T-AT}}} \right)/{\text{dt}} = {\text{k}}_{{{{\rm AT}}}} \cdot \, \left[ {{\text{AT}}} \right]_{{\rm t}} \cdot \left[ {{\text{T}}_{{\rm free}} } \right]_{{\rm t}} $$

2 $$ {\text{d}}\left( {{\text{T-}}{\upalpha }_{2} {\text{M}}} \right)/{\text{dt}} = {\text{k}}_{{{\upalpha }2{\text{M}}}} \cdot \, \left[ {{\upalpha }_{2} {\text{M}}} \right]_{{\rm t}} \cdot \, \left[ {{\text{Tfree}} } \right]_{{\rm t}} $$

3 $$ {\text{d}}\left( {{\text{T-MS}}} \right)/{\text{dt}} = {\text{k}}_{{\rm MS}} \cdot \, \left[ {{\text{T}}_{{\rm free}} } \right]_{{\rm t}} $$

4 $$ - d\left( {{\text{T}}_{{\rm free}} } \right)/{\text{dt }} = {\text{k}}_{{\rm AT}} \cdot \, \left[ {{\text{AT}}} \right]_{{\rm t}} \cdot \, \left[ {{\text{T}}_{{\rm free}} } \right]_{{\rm t}} + {\text{ k}}_{{{\upalpha }2{\text{M}}}} \cdot \, \left[ {{\upalpha }_{2} {\text{M}}} \right]_{{\rm t}} \cdot \, \left[ {{\text{T}}_{{\rm free}} } \right]_{{\rm t}} + {\text{ k}}_{{\rm MS}} \cdot \, \left[ {{\text{T}}_{{\rm free}} } \right]_{{\rm t}} $$

Additionally, thrombin dynamics analysis was used to determine prothrombin conversion curves from TG test data and the model of thrombin inactivation, as described by Eqs. (5 and 6)^11^.

5 $$ {\text{d}}\left( {\text{T}} \right)/{\text{dt }} = \, - {\text{d}}\left( {\text{P}} \right)/{\text{dt}} - {\text{d}}\left( {{\text{T-inh}}} \right)/{\text{dt}} $$

6 $$ - {\text{d}}\left( {\text{P}} \right)/{\text{dt }} = {\text{ d}}\left( {\text{T}} \right)/{\text{dt }} + {\text{ k}}_{{\rm AT}} \cdot \, \left[ {{\text{AT}}} \right]_{{\rm t}} \cdot \, \left[ {\text{T}} \right]{\text{t }} + {\text{ k}}_{{{\upalpha }2{\text{M}}}} \cdot \, [{\upalpha }_{2} {\text{M]}}_{{\rm t}} \cdot [{\text{T}}]_{{\rm t}} $$

The total inhibitory potential of each plasma was quantified by the thrombin decay constant (TDC) and prothrombin conversion curves were quantified by.

1. The total amount of prothrombin converted during the TG test (PC_tot_);
2. The maximum rate of prothrombin conversion (PC_max_);
3. The amount of thrombin-AT complexes formed (T-AT); and
4. The amount of thrombin- α_2_M complexes formed (T-α_2_M).

### In silico experimentation

The computational model for thrombin inactivation by AT and α_2_M was used to perform in silico experiments that show the effect of plasma AT reduction on TG in haemophilia A patients. The in silico TG curve (∫d(T)/dt) was computed using thrombin dynamics Eqs. (1)–(6) while the plasma level of antithrombin ([AT]_0_) was varied in silico in the thrombin dynamics algorithm for thrombin inactivation (d(T-inh)/dt).

As AT levels are know to influence the course of prothrombin conversion indirectly, the individual prothrombin conversion curves were transformed to accommodate the effect of AT lowering^33^. We have shown previously that although the plasma AT level does not influence the total amount of prothrombin conversion (PC_tot_), it does affect the maximal rate of prothrombin conversion (PC_max_) dose-dependently^33^. To correct for this effect we fitted the relationship between plasma AT level and PC_max_ in experimentally determined AT dose–response curves and transformed the prothrombin conversion curves obtained in the hemophilia A patients accordingly for each plasma level of AT.

The input of the model was the previously determined prothrombin conversion curve (d(P)/dt) of each individual patients and. Each individual prothrombin conversion curve together the patients variable AT level and α_2_M and fibrinogen levels were used to calculate a series of 14 TG curves, at 100% AT (of the patients original plasma level), 90%, 80%, 70%, 60%, 50%, 40%, 30%, 25%, 20%, 15%, 10%, 5% and 2.5%. Each in silico generated TG curve was quantified by the peak height, ETP and velocity index were derived from the in silico calculated curves. The TG parameters derived from in silico TG curves obtained at the original AT level of each patient did not differ from the original experimentally determined TG parameters in the individual patients.

### Statistics

GraphPad Prism (was used to perform statistical analyses. A Mann–Whitney U or Kruskal–Wallis test was performed to test for differences between groups, and correlations were assessed using the Spearman’s correlation coefficient.

## Data Availability

The datasets supporting this study are available from the corresponding author (r.delaat@thrombin.com) on reasonable request.
